# Massive Deposition and Accumulation of Hydroxyapatite Crystal after Total Hip Arthroplasty: A Case Report

**DOI:** 10.1155/2013/305180

**Published:** 2013-02-07

**Authors:** Shin Yamada, Koji Nozaka, Hidetomo Saito, Hiroaki Kijima, Hiroshi Tazawa, Yasusi Takasaki, Yoichi Shimada

**Affiliations:** ^1^Department of Orthopedic Surgery, Akita University Graduate School of Medicine, 1-1-1 Hondo, Akita 010-8543, Japan; ^2^International Center for Research and Education on Mineral and Energy Resources, Akita University, Japan

## Abstract

We presented a case in which massive hydroxyapatite accumulation was observed around the artificial hip joint. A 66-year-old female showed a massive accumulation of fluid in and around the hip joint, and milk-like aspirate was obtained. Her aspirate culture was negative, and sediment analysis by X-ray diffraction showed that its component was hydroxyapatite. Since pain was mild, the patient was treated conservatively. To our knowledge, this is the first case in which liquid hydroxyapatite (milk of calcium) was accumulated around the artificial hip joint.

## 1. Introduction

Hydroxyapatite crystal deposition disease (HADD) is characterized by deposition of crystal of hydroxyapatite (HA) in and around the joints [1, 2]. It is known that HA crystal induces arthritis and periarthritis, but its mechanism has not been clarified yet. HA crystal is most frequently observed around the shoulder joint, and it is usually deposited in the soft tissue or bursa around the joints [1–4]. In this paper, a case in which a massive milk-like fluid was accumulated in the articular cavity and in the bursa around the prosthesis after total hip arthroplasty (THA), and HA crystal could be identified by X-ray diffraction analysis (XRD), was presented.

## 2. Case Presentation

A 66-year-old female complained of left hip pain. She was operated bilateral THA using AML system (Depuy Co., Ltd.) due to hip osteoarthritis at the age of 52 years in 1994. She developed interstitial pneumonia at the age of 63 years and was diagnosed as having mixed connective-tissue disease (MCTD) at the age of 64 years. She had taken oral prednisolone (15–20 mg per day) after the diagnosis. In addition, preparations of calcium, vitamin D, and vitamin K2 were taken for the treatment of osteoporosis. Thirteen years after THA, she visited an orthopedist at previous facility and was diagnosed as having osteolysis around the left artificial hip joint and was introduced to our facility.

**Figure 1:**
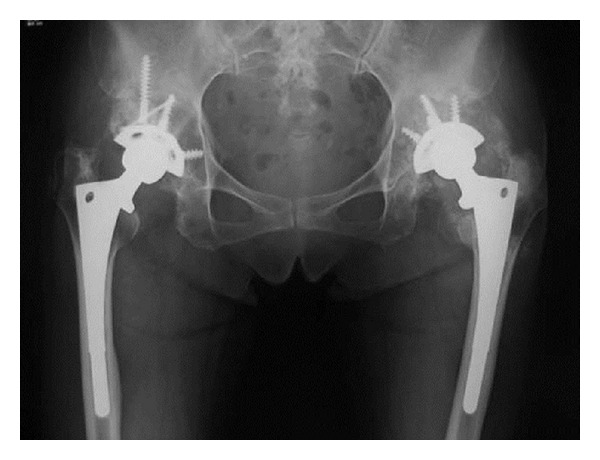
X-ray findings at the initial diagnosis.

**Figure 2:**
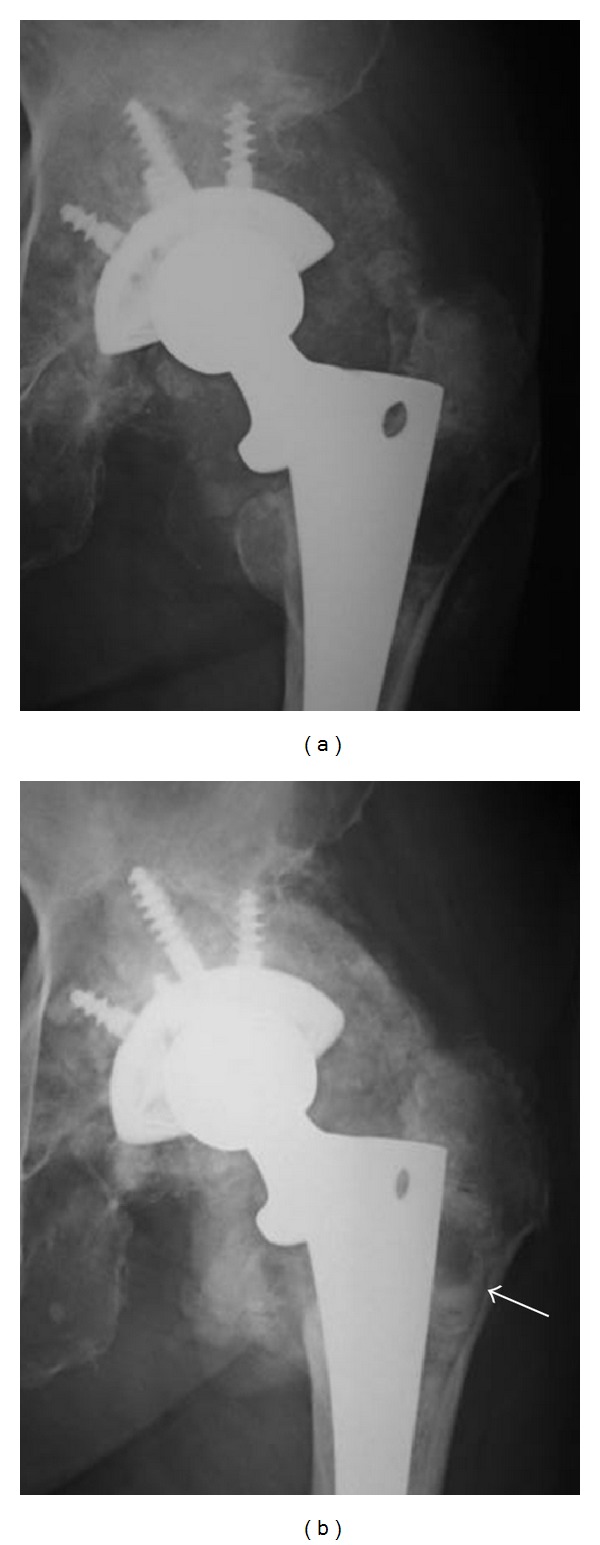
Comparison of X-ray findings at the initial diagnosis (a) and 1 year later (b). Calcification around the left hip prosthesis was observed. The arrowhead indicates niveau formation in the greater trochanter marrow cavity.

**Figure 3:**
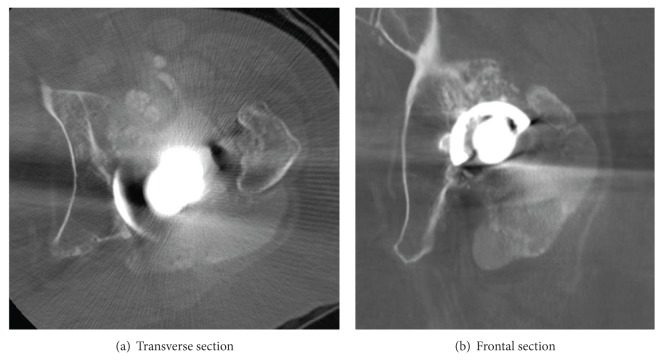
CT findings. Multiple calcified lesions and accumulated fluid in the capsule and in bursa were observed.

**Figure 4:**
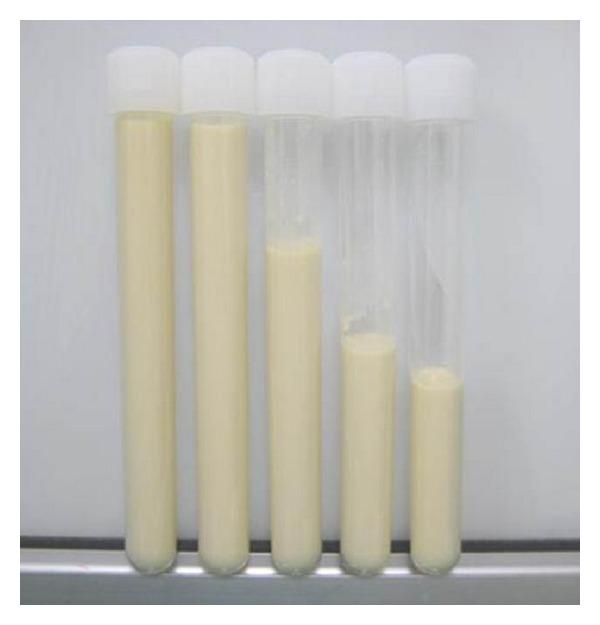
State of the puncture: white milky turbid solution.

**Figure 5:**
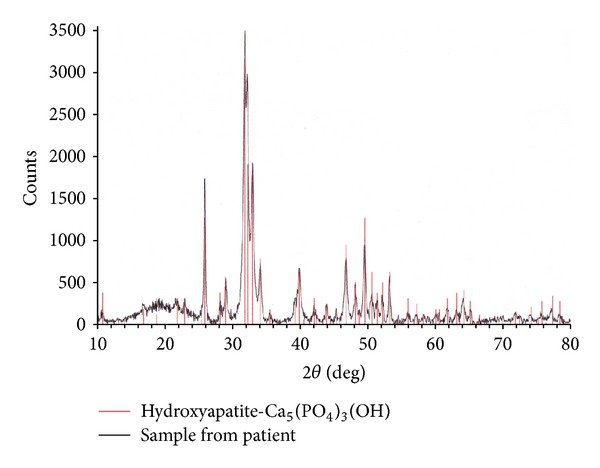
Result of X-ray diffraction analysis (XRD).

X-ray photograph taken at the first visit of our facility revealed polyethylene abrasion of the left artificial hip joint, and osteolysis of the left acetabulum and femur (Figure 1). She had been followed up as an outpatient, but findings suggesting calcification around the left artificial hip joint were observed one year later. In addition, niveau formation suggesting fluid accumulation was observed in the greater trochanter hollowed due to bone lysis (Figure 2). CT showed a calcified lesion and massive fluid accumulation in the articular cavity and in the bursa (Figure 3). Blood biochemical examination showed normal CRP, Ca, P, PTH, intact PTH, and ALP. Milky fluid of 120 mL was collected thorough joint puncture (Figure 4). Microscopic examination rarely showed cell components, and no urate crystals or calcium pyrophosphate dihydrate (CPPD) was identified. Bacteria were not detected by a culture test. In X-ray fluorescence analysis (XRF) of the aspirate, calcium/phosphorus (Ca/P) molar ratio was 1.69, which was similar to that of the typical HA (1.67). In X-ray diffraction analysis (XRD), the diffraction pattern of samples was almost the same as that of authentic HA (Figure 5).

## 3. Discussion

HADD is characterized by HA deposition in and around the joints. It can occur in any joints, but it is most frequently observed in the shoulder joint [2, 5]. When rapid precipitation and outflow to the surrounding tissue of crystal occur, crystal-induced acute inflammation is induced. Macroscopic findings of the aspirate in HADD patients are expressed as “milky” or “cheesy.” Pathological conditions under which accumulation of massive joint fluid including HA crystal and extensive rotator cuff tear and articular destruction are induced are known as Milwaukee shoulder [3, 6, 7]. It is very rare that accumulation of massive fluid including HA occurs in the joints other than the shoulder joint.

To our knowledge, a case of HADD developed after THA has not been yet reported. In our case, the findings of collapse and communication with the surrounding bursa of the pseudocapsule, bone atrophy of the acetabulum and femur, and accumulation of massive fluid including HA were pretty similar to those of Milwaukee shoulder. However, a macroscopic finding of the aspirate in our case (milky) differed from that of Milwaukee shoulder (serosanguinous) [3]. This suggested that HA concentration in our case was extremely high.

Whether the artificial hip joint was involved in HADD occurrence was not clear in our case, but it was interesting that the lesion was developed only in the left side whose polyethylene abrasion and bone lysis were remarkable even though the THA was performed bilaterally using the same devices around the same time. There was a possibility that the abrasion powder of polyethylene stimulated macrophages, and the released cytokine activated the osteoclasts to induce bone lysis, which might be a pathogenesis. However, cases of complication of bone lysis and HADD after THA have not been previously reported, and only the above could not be a pathogenesis. It is well known that HADD is frequently associated with connective tissue diseases and renal failure [1]. In our case, both a connective tissue disease and bone lysis around the left artificial hip joint were thought to cause HADD.

## 4. Conclusions

In this paper, a case was presented in which a massive milky fluid was accumulated around the artificial hip joint accompanied by bone lysis, and HA crystal was identified by XRD. Both a connective tissue disease and bone lysis due to polyethylene abrasion were thought to cause HADD.
